# A Globally Generalized Emotion Recognition System Involving Different Physiological Signals

**DOI:** 10.3390/s18061905

**Published:** 2018-06-11

**Authors:** Mouhannad Ali, Fadi Al Machot, Ahmad Haj Mosa, Midhat Jdeed, Elyan Al Machot, Kyandoghere Kyamakya

**Affiliations:** 1 Department of Smart Systems Technologies, Alpen-Adira University, Klagenfurt 9020, Austria; Ahmad.HajMosa@aau.at (A.H.M.); midhat.jdeed@aau.at (M.J.); kyandoghere.kyamakya@aau.at (K.K.); 2 Research Center Borstel—Leibniz Center for Medicine and Biosciences, Borstel 23845, Germany; falmachot@fz-Borstel.de; 3 Carl Gustav Carus Faculty of Medicine, Dresden University of Technology, Dresden 01069, Germany; Elyan.Al-Machot@uniklinikum-dresden.de

**Keywords:** emotion recognition, classification, dynamic calibration, cellular neural networks (CNN), physiological signals

## Abstract

Machine learning approaches for human emotion recognition have recently demonstrated high performance. However, only/mostly for subject-dependent approaches, in a variety of applications like advanced driver assisted systems, smart homes and medical environments. Therefore, now the focus is shifted more towards subject-independent approaches, which are more universal and where the emotion recognition system is trained using a specific group of subjects and then tested on totally new persons and thereby possibly while using other sensors of same physiological signals in order to recognize their emotions. In this paper, we explore a novel robust subject-independent human emotion recognition system, which consists of two major models. The first one is an automatic feature calibration model and the second one is a classification model based on Cellular Neural Networks (CNN). The proposed system produces state-of-the-art results with an accuracy rate between 80% and 89% when using the same elicitation materials and physiological sensors brands for both training and testing and an accuracy rate of 71.05% when the elicitation materials and physiological sensors brands used in training are different from those used in training. Here, the following physiological signals are involved: ECG (Electrocardiogram), EDA (Electrodermal activity) and ST (Skin-Temperature).

## 1. Introduction

Emotion is a complex phenomenon which involves various physical structures. It plays an important role in decision-making, behavior and other social communication. The ability to understand and recognize human emotion has been identified as one of the key focus areas listed by research groups in different fields of intelligent systems [1] such as safe driving [2], health care [3], social security [4], multimedia digital entertainment [5] and other fields. Moreover, human emotions can be extracted from measured appropriate physiological sensor date. Most researchers in the field of emotion recognition have focused on the analysis of data originating from a single sensor, such as audio (speech) or video (facial expression) data [6,7]. Lately, many studies in the emotion recognition field have started to combine multiple sensors data to build a robust emotion recognition system. The main target of using the fusion of multiple sensors is that humans use a combination of different modalities in our body to express emotional states during human interaction [8]. The human modalities are divided into audiovisual (facial expression, voice, gesture, posture, etc.) and physiological (respiration, skin temperature, etc.) [8]. However, the recognition of the emotional state is still a complex scientific challenge. One of the main difficulties is that the emotion-relevant signal patterns may widely differ from person to person or from a specific situation to another. Moreover, it is hard to find the exact correlation between the classes (patterns) due to the problem of the precise definition of emotions and their meanings [9]. Additionally, emotions are complex sets of interactions among subjective and objective factors, mediated by neural/hormonal systems in the physiological system of the subject, which can be affected by experiences to rise the arousal, pleasure and displeasure and consequently lead to behaviors that are often expressive, goal-oriented and adaptive. Thus, they might differ and depend on age, culture and many other social issues. Based on the previous points, computers can be made to understand human emotions by capturing these modalities, extracting a set of useful features from them and fusing those features in order to infer an accurate emotional state [9]. There is a growing number of sensors that can capture various physical manifestations of emotion: video recordings of facial expressions [10], vocal inflection changes [7], recording of brain waves using Electroencephalogram EEG [3], skin-surface sensing of muscle tension [11], electrocardiogram (ECG) [12], electrodermal activity (EDA) [13], skin temperature (ST) [14], etc.

As a result, the recognition of human emotions has reached promising results. However, such a high performance is mostly related to subject-dependent cases and not for subject-independent scenarios. Thus, due to the challenges in this perspective, which make the recognition more complex, it is required from the research community to focus on developing universal systems that can detect human emotions generally using once pre-trained machine learning models. Therefore, in this paper, we try to overcome such challenges.

Consequently, the importance of this work is due to the fact that developing a universal emotion recognition system is challenging, which can be trained locally once and after that tested considering different data that are collected based on different lab settings. In other words, where subjects, elicitation materials and physiological sensors brands are different from the ones involved in the initial training.

This paper is organized as follows: in Section 2, an overview of emotions and related works are provided. Section 3 is about the physiological signals involved in this work. Then, Section 4 introduces the overall architecture of our proposed system. Section 5 describes the research methodology. The emotion recognition performance and a benchmark evaluation are then presented in Section 6. Finally, the conclusion of this work is given in Section 7.

## 2. Background

### 2.1. Definition of Emotion

The emotion is a complex concept involving two components [15]:

- Subjective experience: several works have categorized emotions into different states, whereby all humans regardless of culture and race can experience them. However, the way of experiencing these emotions is highly subjective [9].
- Emotion expressions: most expressions are observable and nonverbal behaviors, which illustrate an affective or internal emotional state. For example, happiness and pleasure can be expressed by a smile, whereby sadness or displeasure by a frown. In general, emotion expressions include human audiovisual activities such as gesture, posture, voice intonation, breathing noise, etc.

Emotions can be classified in various ways. The most applied two models for emotion classification are the “discrete emotion model” proposed by Ekman [16] and the “emotion dimensional model” proposed by Lang [17]. The discrete emotional model categorizes emotions into six basic emotion states: happiness, sadness, surprise, anger, disgust and fear [16]. These emotions are universal, biologically experienced by all humans and widely accepted in this research field. On the other hand, the dimensional model assumes that the emotions are a combination of several psychological dimensions. The most well-known dimensional model is the “valance-arousal dimensional model”. The valance represents a form of pleasure level and ranges from negative to positive. However, the arousal indicates the physiological and/or psychological level of being awake and ranges from low to high [11].

### 2.2. Related Works

Several emotion recognition studies have been conducted in the field of human–machine interaction using different physiological signals. Initially, those studies used some emotion elicitation materials such as video, images or music to elicit certain emotions from a subject(s), while the related physiological measures have been recorded. Then, meaningful features are extracted from these physiological measures and classified into emotional states using diverse classifiers.

For the purpose of building reference databases to be used for both training and validation testings, different works used images [18,19], music [11], cognitive tasks [20], complicated mathematical problems [21], movie clips [22], questionnaires [9] and other methods to elicit emotions. The benefit of using images is that they are easy and fast to apply and can also be self-reported by participants. However, the disadvantages of this method are that the images might not be able to evoke some strong emotions and the time for stimulating emotion is too short.

Lin et al. [11,23] have applied music to stimulate emotions and used an electrocardiogram (ECG), respiration, skin conductance and electromyogram signals to identify the induced emotions. In general, the advantages of using music to induce emotions is simple, highly standardized and emotions develop thereby over time (15–20 min). On the other hand, the disadvantages of this later approach are related to participants music taste, which might influence the experienced emotions. Therefore, this method gives only the moods (positive or negative) [24]. Moreover, Wen et al. [22,25] have used a set of short movies as an emotion elicitation method, which is a rich instrument to induce strong emotions (love, anger, fear, joy, etc.), which can also be self-reported by participants. The drawback is, however, that it is necessary to extract particular periods of interest from the movie. Additionally, due to the fact that emotions are considered as evanescent phenomena, any delay between emotion activation and its assessment by an experimenter can introduce an error in the measurement [24].

After gathering the referenced (annotated) affective data, many feature extraction and classification techniques have been used in literature. First works have mostly focused on subject-dependent approaches, where the emotion recognition system is performed only on one subject and the system needs to be retrained in order to perform well on another subject. Currently, the focus has shifted more towards subject-independent approaches, where  the emotion recognition system should perform well on different subjects without the need to retrain the model, i.e., the system is tested with unknown physiological signals of other persons. Table 1 lists a short review of previous works in emotion recognition using physiological signals. The table illustrates for each work, (a) the stimuli that were used for emotion elicitation, (b) physiological signals that were measured, (c) which emotional states were recognized, (c) the number of subjects who participated in the experiments, (d) which features and classification methods were applied, and (e) the respective performance of the recognition approaches used.

We can observe that, regarding the subject-dependent approaches, Haag and Goronzy [18] have extracted, as features, both running mean and running standard deviation slopes from five physiological signals and reached the highest accuracy values of 96.58% for subject-dependent cases by involving a neural network classifier for recognizing three arousal levels (high, medium and low). Kim and Andre [11] proposed an approach based on a Linear Discriminant Analysis (LDA) classification scheme for classifying four emotions (joy, anger, sad and pleasure) involving four physiological signals and reached 95% accuracy for subject-dependent cases, but the accuracy decreased to 70% for subject-independent ones. Moreover, Lisetti and Nasoz [14] could reach 91.7% classification subject-dependent accuracy for six emotions (amusement, frustration, anger, fear, sadness and surprise). On the other hand, for subject-independent approaches, the highest accuracies 99.5% was reached by [20] for recognizing one emotion (stress) using classification method based on fuzzy logic. WanHui et al. [12] could reach 86% subject-independent accuracy for two emotions (joy and sadness).

In our previous work [13], we have developed a subject-independent emotion recognition approach based on cellular neural networks (CNN) and we could thereby reach 82.35% of accuracy for four emotional states (High/Low arousal and High/Low valence). Overall, one should notice the following core building bricks or aspects of relevance of/for a robust and reliable emotion recognition system: the sensors used, the number of subjects involved in training and testing, the number emotional states to be detected, the stimuli used for inducing emotions, the features extraction and selection, and the classification method.

### 2.3. The Present Work

In this work, we are proposing a universal robust emotion recognition system, which should perform well in environments that are different from the ones of its initial training (i.e., different subjects, different elicitation instruments/contexts, and different physiological sensors brands). The most well-known features in the literature are extracted from three physiological signals—EDA, ECG and Skin temperature—in order to classify the emotional states of different subjects. More details about the features extraction are presented in the Section 4.3.

For a robust subject-independent classification of the induced emotional states for different test subjects, this paper does involve a so-called “Adaptive CNN”. The adaptive CNN is presented in Section 4.5 further below in this paper. Regarding the training set, a publicly available emotion reference database, MAHNOB [26], is used for training the proposed system, whereby, however, data collected from our special experimental setting are used for testing purposes. Moreover, in order to ensure a robust performance while testing in different testing contexts/environments, an automatic calibration model is introduced for the purpose of calibrating the testing data with respect to the ones involved in the initial training process.

Therefore, our proposed system consists of two major novel contributions for handling the concern of ensuring a robust and universal subject-independent human emotion recognition. The first contribution is:

- The automatic features’ calibration for an adaptive adjustment of the extracted features by translating them toward the correlated subject in the training set. Here, we use the collaborative filtering concept of [27] to calculate the adjustment weight of the extracted features from a new subject by finding its most correlated subject from the training data.
- A novel machine learning model based on Cellular Neural Networks (CNN) that delivers promising results. Here, we improved the performance of the CNN processor by using a hyperbolic tangent sigmoid transfer function [28] as output nonlinear function of the CNN states and the echo-state network ESN [29] paradigm for an efficient training of the CNN processor model.

## 3. Physiological Signals Involved in This Study

Different types of physiological signals can be measured from human beings by electronic measurement or sensor artefacts. After appropriate processing, information related to health and/or emotion can be extracted from those signals. In this study, the following physiological signals EDA, ECG and ST are considered due to the better performance achieved by using just these signals and the corresponding physiological sensors are more comfortable to attach to a human body.

- Electrodermal Activity (EDA): It refers to skin conductivity (SC) that basically measures the skin’s ability to conduct electricity, whereby the conductivity increases if the skin is sweaty. During the experience of physical arousal, the central nervous system is activated and the sweat is produced in the endocrine glands, which measurably changes the conductivity of the skin [30].
  EDA consists of a slowly changing part called Skin Conductance Level (SCL), which is overlaid by other short and fast conductance changes called phasic components. The phasic components can be separated into two different types. The first one is the Skin Conductance Response (SCR), where the peak occurs in reaction to a stimulus. The second one is the Non-Specific Skin Conductance Response (NS.SCR), which is empirically very similar to SCR, but, however, occurs spontaneously without any stimulus [6]. In our study, the EDA signals are measured with a sampling rate of 4 Hz using a wearable wireless device (*Empatica*—*E*4 [31]) placed on the human wrist.
- Electrocardiogram (ECG): It refers to a measurement setting that measures the electrical activity of the heart over a period of time. In general, ECG signals consist of three main waves. The first wave is the P wave, which indicates the depolarization of the atrium. The second wave is the QRS wave, which corresponds to the start of ventricular contractions. After the ventricles have stayed contracted for a few milliseconds, the third wave T appears. This wave occurs when the ventricular repolarizes [32]. The wearable wireless Bioradio™ device [33] (Great Lakes NeuroTechnologies, OH, USA) is used to measure the ECG signal with three electrodes (plus one ground electrode) placed on the body at a sampling rate of 500 Hz.
- Skin Temperature (ST): The skin temperature is recorded with an optical infrared thermometer. The ST signals are measured with a sampling rate of 4 Hz using a the wearable wireless device (*Empatica*—*E*4 [31]), which also incorporates the EDA measurement artefacts and is placed on the human wrist.

## 4. Research Methodology

This section provides a description of the overall research methodology, a comprehensive presentation of the physiological reference dataset MAHNOB used for training, and the presentation of our lab dataset used for final testing and validation of the proposed emotion recognition system developed in this study. Furthermore, a full description of the involved feature extraction methods of each physiological signal is provided. Moreover, our proposed feature calibration model and the adaptive CNN classifier are presented.

**Figure 1:**
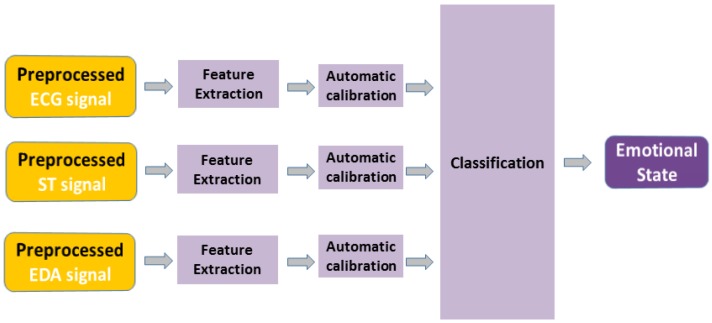
The general architecture of the proposed emotion recognition system.

The overall architecture of our proposed system is illustrated in Figure 1. After the preprocessing stage of all involved physiological signals, the features’ extraction step (involving best related concepts from the relevant state-of-the-art) is explained. Moreover, the novel “Features’ Calibration” model to improve the performance of the subject-independent classification is explained. At the end of this section, our proposed Cellular Neural Network (CNN) based classification model is described and demonstrated. It should be noticed that this CNN classifier will be benchmarked (see Section 5) with other relevant competing classifiers from the state-of-the-art.

### 4.1. Data Collection and Experiment Procedure

In the frame of this study, a publicly available reference dataset of data signals is used for training, testing and an initial validation of the developed emotion detection approach. For the final validation of the system, however, a new own special set of emotion analysis experiments (on new real persons) has been conducted for a last comprehensive stress-testing for a final validation. The public dataset used is called MAHNOB and has been collected by Soleymani et al. [26]. It includes different physiological signals from 30 young healthy adult participants between 19 to 40 years, 17 female and 13 male. All signals were downsampled to 256 Hz and the trends (overall patterns that are not intrinsic to the data) of both ECG and galvanic skin response (GSR) signals were removed [26]. Each of the involved subjects watched 20 emotional video clips and performed a self-assessment of his/her related respective degree of both valence and arousal by using the so-called Self-Assessment Manikins (SAM) questionnaire [34]. Moreover, in our own data collection experiments for emotion analysis (used for the final system validation), six healthy volunteers were involved. All were healthy subjects (three male and three female) in the age ranges of 19 to 30 years. The identity of subjects is not known (i.e., anonymous) and they were not allowed to consume any stimuli like drugs, alcohol, or caffeine before the experiment. At first, a suitable interface has been implemented for the automated projection of the emotion-related videos and the corresponding self-assessment of the emotions each time a video is shown. In a preliminary study, 100 video clips containing movie scenes or short videos from youtube.com were manually selected and showed online to anonymous participants. The participants were asked to self-assess their respective related emotion after watching each video by reporting the respectively experienced arousal and valence levels on a nine-points scale using Self-Assessment Manikin (SAM) [34], on one side, and also the experienced respective universal discrete emotions: happiness, surprise, anger, disgust, sadness, and fear. Seventeen video clips from the clips that received the highest number of tags in different emotional states were chosen based on the preliminary study. The selected videos were kept as short as possible (between 3 and 5 min) in order to avoid multiple emotions reacting to the same stimulus from occurring. Hereby, one has taken into account the information that emotion specialized psychologists recommend video lengths in the range of one minute to a maximum of ten minutes for the elicitation of a single emotion [35,36].

The core of our own above-mentioned data collection experiment starts by providing a set of instructions to the participant for him to understand the experiment procedures (i.e., the steps) and the meaning of the different scales used for self-assessment. After the instructions have been understood by the participant, the sensors are placed on him and the sensors are checked to see whether they function well. Then, the participant is led into the experiment room. At the beginning, the participant performs a trial session to get familiar with the technical system supporting both the emotion elicitation and the related data recording. In this trial phase of the process, a short video is shown, followed by a trial self-assessment by the participant. After the trial phase is judged positive, the recording of the physiological signals is activated and the experiment session starts when the participant clicks the START button on the computer screen. In the session, 17 video clips are presented to the participant in 17 sequences, each consisting of the following steps: (a) a 5 s counter in order to the get the participants’ attention; (b) 3 to 5 min display of the video clip; and (c) the self-assessment by the participant of the respectively experienced emotion by giving related subjectively perceived levels or values of the following parameters: arousal, valence, liking, dominance, and the discrete emotion state.

### 4.2. Data Synchronization and Target Classes

The physiological data generated by our experiment are synchronized by using the session starting time as a reference timestamp. Moreover, the signals from the reference database MAHNOB and the signals from our experiment have different sampling rates. To correct this fact, all signals have been re-sampled as the following: ECG is re-sampled to 256 Hz, EDA re-sampled to 4 Hz and ST re-sampled to 4 Hz. Regarding the target classes, in this paper, we have mapped the scales (1–9) into two levels (classes) of each valence and arousal state according to the SAM ratings. The valence scale of (1–5) was mapped to ”Low-Valance” and (6–9) to ”High-Valance”, respectively. The arousal scale of (1–5) was mapped to ”Low-Arousal”, and (6–9) to ”High-Arousal”, respectively.

### 4.3. Feature Extraction

In this paper, the most commonly used features from the relevant state-of-the-art are taken and used as such, as we do not intend to create new features. This is the case for each physiological signal involved/considered in this study.

From each of the three involved physiological signals, we have extracted 12 EDA features, eight ECG features and five skin temperature features.

#### 4.3.1. EDA Features

The EDA signal consists of two parts: the slow changing part is called the skin conductance level (SCL) and the phasic components part is called skin conductance responses (SCRs) (see Section 3). Statistical measures extracted from the SCL analysis have been found to be well-correlated with emotion [37]. Here, the following statistical features are used: the mean, the standard deviation, the maximum, the minimum, the root mean square, the mean of the first derivation, the mean of the second derivation, and the mean of negative slope. For SCR analysis, we use, as features, the SCR occurrences rate from the very low frequency band (0–0.1 Hz), the response latency of the first significant SCR, the sum of SCR amplitudes, and the sum of SCR areas [37].

#### 4.3.2. ECG Features

ECG features are extracted from both the time-domain and frequency domain. From the time-domain, the following statistical features are calculated directly from the ECG signals: the mean, the standard deviation of the beat-to-beat interval (NN interval), the root mean square of differences of successive NN intervals, the number of successive differences that are greater than 50 ms, and the percentage of total intervals that successively differ by more than 50 ms [38].

From the frequency domain, we use, as features: the average power of the low frequency range (0.04–0.15 Hz), the average power of the high frequency band (0.15–0.4 Hz), and the ratio of the power within the low frequency band to that within the high frequency band [38].

#### 4.3.3. Skin Temperature Features

The standard statistical feature moments (mean, max, and STD) are calculated from the skin temperature signal.

### 4.4. Automatic Calibration Model

The physiological expressions of emotion might differ and depend on age, culture and many other social issues. This makes subject-independent emotion recognition a very challenging task. Moreover, the challenge becomes even more complex whenever emotion recognition is applied on subjects in different environments, where both elicitation materials/contexts and physiological sensors brands are much different from the ones involved in the training phase. To overcome this problem, this work tries to find out whether the test subject has possibly similar physiological reactions like a certain subject from the training set. By similarity, it is meant that the two subjects have almost the same physiological reaction in response to a given emotion related stimuli. Hence, after finding the most correlated subject from the training set, we transform the features set of the test subject towards the features space of the equivalent correlated subject from the training set. In this way, the features of the new test subject are calibrated from the perspective of the training environment.

Practically, from the training set, the data of each subject is clustered to a specific number of clusters using an unsupervised learning method. The centroids of each subject are kept to be used during calibration. Through testing, the features set of a new test subject is calibrated by shifting his features set towards its most correlated subject from the training data; this is based on the idea of and inspired from the so-called collaborative filtering [27]. Here, the correlation between the features vector of the new subject and the centroids of the training subjects is calculated. Then, from the correlation results, the centroid with the best correlation is selected and the feature set of the new subject is shifted towards this selected centroid. In general, the proposed model consists of two phases, a preparation and an online phase:

#### 4.4.1. Preparation (i.e., Offline) Phase

In this phase, we may determine reference points from the features of each subject from the training set. Those reference points could then be used in the online phase to correlate them with the features of a new test subject. Finding meaningful reference points for the training subjects is the key step to calibrating the new features from test subjects robustly. Considering the representative (mean centroid) of the subject’s features as a reference point is not useful because the subject’s features are representing different emotional states and the center might represent just one emotional state. This would lead to a wrong calibration. Moreover, selecting the centroids of the emotional classes as reference points (in our case, we have four emotional states, which leads to four centroids for each subject), is not accurate because the new features set (from a test subject) would be shifted towards a specific emotion level.

However, to solve this issue, we propose an unsupervised clustering method to select different centroids from each subject. Here, the k-means clustering [39] is considered. After selecting the initial centroids using K-means++ algorithm [40], the Cosine distance function is used to calculate the distance between the initial centroids and the feature sets (see Equation (1)):    

(1) $$\begin{array}{c} d\left(x,c\right)=1-cos\left(x,c\right)=1-\frac{x.c}{\left|x||c\right|}, \end{array}$$

where *x* is an observation of the feature sets, *c* is the initial centroid and d(x,c) is the Cosine distance.

Furthermore, the number of clusters has a big impact on the overall performance of our proposed system. Choosing the correct number of clusters is often ambiguous and depends on the shape and scale of the distribution of points in a data set. The Gaussian-means (G-means) algorithm [41] is involved to determine the number of clusters. The G-means algorithm starts with a small number of k-means centers (start with one cluster), and then keeps splitting clusters until the data assigned to each cluster has a Gaussian distribution (see Algorithm 1).

**Algorithm 1** G-means algorithm.
1:**Input:***X* the set of data and α confidence level = 90% (Gaussian)  2:**Output:** The new set of centers *C* (using K-means++ [40])  3:Given *C* the initial set of centers  4:C←k−means(C,X)  5:**Let**$\left\{x_{i}|class\left(x_{i}\right)=j\right\}$ be the set of the data assigned to center $c_{j}$  6:**Check** if each $\left\{x_{i}|class\left(x_{i}\right)=j\right\}$ follow a Gaussian distribution (at confidence level α)  7:**if** The data follow a Gaussian distribution **then**  8:    Keep $c_{j}$  9:
**else**
10:    replace $c_{j}$ with two centers (using K-means++ [40]).  11:**end if** 12:**Repeat** from step 5 until no more centers are added.


Finally, the total number of clusters after applying the G-means algorithm is between 8 and 14 clusters per subject. In addition to clusters centroids, the standard deviation of each cluster is calculated to be used later in the online phase to tune the feature transforming.

#### 4.4.2. Online Phase

In the online phase, after a signal of a test subject has been pre-processed and the features extraction process is done, we try to find the correlation between the new features set and the calculated cluster centers of each subject in the training set from the offline phase. One popular measure of similarity in Collaborative Filtering is the Pearson’s correlation coefficient. Given the features vector *X* and the centroid of one cluster *C* (here, the features vector and the centroid have the same length *N*), the Pearson’s correlation coefficient *r* between *X* and *C* is given in Equation (2) [42]:

(2) $$\begin{array}{c} r_{x,c}=\frac{\sum_{i=1}^{N}\left(X_{i}-\mu_{x}\right)\left(C_{i}-\mu_{c}\right)}{\left(N-1\right)\sigma_{x}\sigma_{C}},\\ where\;\;\mu_{x}=\frac{\sum X}{N}\;\;,\;\;\mu_{c}=\frac{\sum C}{N},\\ \sigma_{x}=\sqrt{\frac{\sum_{i=1}^{N}{\left(X_{i}-\mu_{x}\right)}^{2}}{N-1}}\;\;,\;\;\sigma_{c}=\sqrt{\frac{\sum_{i=1}^{N}{\left(C_{i}-\mu_{c}\right)}^{2}}{N-1}}, \end{array}$$

where μ is the mean, σ is the standard deviation and *N* is the length of the features vector and of the cluster centroids.

There, the result of Pearson’s correlation varies between −1 and 1, where  −1 means negative correlation, 0 means no correlation, and 1 means positive correlation. Next, the center of the highest absolute correlation |r| with the features vector of a new subject is selected to calculate the distance *d* between the center and the new features vector using Euclidean distance (see Equation (3)):

(3) $$\begin{array}{c} d_{C,V}=\sqrt{\sum_{i=1}^{N}{\left(c_{i}-v_{i}\right)}^{2}}, \end{array}$$

where *C* and *V*, respectively, are the center and the new features vector of the length *N*. Moreover, to guarantee that the features vector of the new subject is not overlapped with the center of the correlated subject during calibration, the distance $d_{C,V}$ is normalized using Equation (4):

(4) $$\begin{array}{c} D_{C,V}=d_{C,V}*J_{n},\\ d_{n}=\frac{D_{C,V}-C}{\sigma_{C}}, \end{array}$$

where *C* is the centroid, $J_{n}=\left(1,1,\ldots ,1\right)$ is a vector of all-ones of the length *N* (same length as the centroid *C*) and $\sigma_{C}$ is standard deviation of the cluster of the centriod *C*.

Finally, the features vector of the new subject is calibrated by translating the features towards the centroid by applying the element wise subtraction between the features vector and the normalized distance $d_{n}$, see Equation (5):

(5) $$\begin{array}{c} D_{n}=d_{n}*J_{n},\\ V_{new}=V-D_{n}. \end{array}$$

### 4.5. Classification

In this chapter, a Cellular Neural Network (CNN) based classification is introduced. The proposed CNN architecture is an improved version of our previous works [2,13]. CNN was suggested by Chua and Yang (1988) [43]. It combines the advantages of Cellular Automata (CA) and artificial neural networks (ANNs) but differentiates by its nonlinear dynamical relation between cells and local connectivity. CNN is a network of adjacent coupled nonlinear cells where the relationship between the connected cells is modelled by a system of differential equations. The general state equation of a CNN cell is given in Equation (6):

(6) $$\begin{array}{c} \frac{dx_{i}\left(t\right)}{dt}=-x_{i}\left(t\right)+\sum_{j=1}^{n}A_{i,j}y_{j}\left(t\right)+\sum_{j=1}^{m}B_{i,j}u_{j}\left(t\right)+B_{i}, \end{array}$$

where $x_{i}\left(t\right)$ is the current system state and $u_{j}\left(t\right)$ is $i_{t}h$ input. $A=(a_{1,1}\ldots a_{n,n})$ is the feedback template, $B=(b_{1,1}\ldots b_{n,m})$ the control template, $B_{i}$ is the cell bias and $y_{i}\left(t\right)$ is the output nonlinear function of the state (see Equation (7)):

(7) $$\begin{array}{c} y_{i}\left(t\right)=\frac{1}{2}\left(\right|x_{i}+1|-|x_{i}-1\left|\right). \end{array}$$

The default nonlinear function (Equation (7)) proposed by Chua is very simple and not sufficient for highly nonlinear problems and multi-class classification. To overcome this drawback, the nonlinear function proposed by Chua in Equation (7) is replaced by hyperbolic tangent sigmoid transfer function [28] (see Equation (8)):

(8) $$\begin{array}{c} y_{i}=tansig\left(x_{i}\right)=\frac{2}{1+e^{-2x_{i}}}-1. \end{array}$$

**Figure 2:**
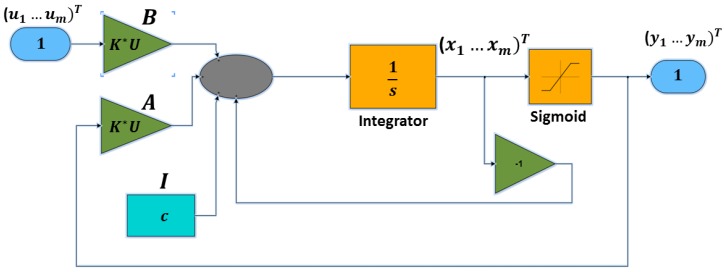
The Cellular Neural Network (CNN) classification model (SimulinkModel A).

Moreover, in order to generate the related CNN output, the differential equation Equation (6), including Equation (8), have to be solved. This is done using Matlab Simulink [44] (R2015b, MathWorks, Natick, MA, USA) (see Figure 2).

In this study, three CNN models are implemented for the three physiological sensors EDA, ECG and ST (CNN model for each sensor). The outputs of each CNN model are combined into the platform output through a linear regression (see Equation (9)):

(9) $$\begin{array}{c} g\left(t\right)=A_{global}Y\left(t\right), \end{array}$$

where $Y=(y_{1}^{ecg-cnn}\ldots y_{n}1^{ecg-cnn},y_{1}^{eda-cnn}$ $\ldots y_{n}^{eda-cnn},y_{1}^{st-cnn}\ldots y_{n}^{st-cnn})$ is a vector of all three CNN outputs and $A_{global}$ is the output linear regression template.

**Figure 3:**
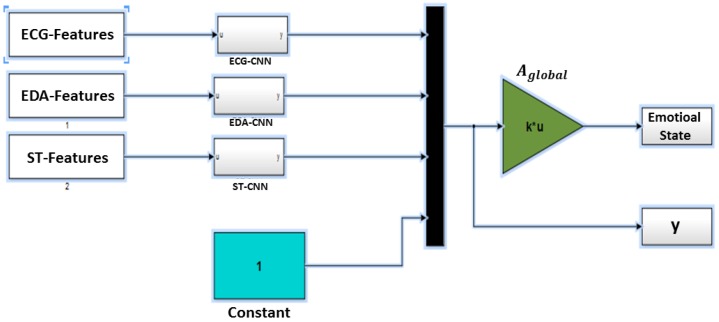
The multi-CNN modal emotional state estimation (SimulinkModel B).

Figure 3 illustrates the related Simulink scheme of the multi-CNN modal emotion state recognition. All input signals are connected to the related CNN model. Each CNN block contains the same scheme with different templates’ configurations. The CNN outputs are multiplexed and biased with a constant to a single vector. The output of the multiplexer is either used to identify the linear regression template $A_{global}$ during the training phase or to determine the estimated emotional state that is used for the testing phase.

#### 4.5.1. Learning Phase

In order for the proposed model to perform properly, a learning procedure (training) needs to be applied on the model. The target of the training is to identify the best feedback templates, the control templates, the biases and the configuration of the CNN state equation. In this phase, we have an optimization problem to deal with. In our previous work, we use the Particle Swarm Optimization (PSO) [45]. The main issue of this method is the increasing time-consumption when dealing with highly dimensional CNN (i.e., with a large number of cells). In order to solve this issue, the echo-state network ESN is used to provide a more efficient CNN approach.

ESN is an innovative approach proposed by Jaeger [29] for training recurrent neural networks (RNN) where it showed excellent performance. In ESN, the state feedback templates, the control templates and biases templates are randomly generated. The random generation process is done as follows:

- A(n×nmatrix) is generated as normally distributed sparse symmetric matrix with N(0,1) and a sparseness measure of 0.5. The resultant matrix is then divided by its own largest absolute eigenvalue. These generating constraints are important to respect the properties of the echo state (sparsity and spectral radius <=1) that give stability for the network as suggested by [46].
- B(n×mmatrix) and I(n×1vector) are generated randomly with a standard normal distribution N(0,1) and scaled by a factor equal to (0.1).

After the CNN templates (ECG-CNN, EDA-CNN and ST-CNN) have been generated, the output layer is finally trained using the Ridge Regression (RR) [46], Equation (10):

(10) $$\begin{array}{c} A_{global}=g\left(t\right)Y{\left(t\right)}^{T}{\left(Y\left(t\right)Y{\left(t\right)}^{T}+\beta C\right)}^{-1}, \end{array}$$

where g(t) is the desired output (see Equation (9)); *C* is the identity matrix and β is the regularization coefficient, which is determined using the cross validation technique. It is necessary to add β in the Ridge Regression to avoid an ill-conditioned problem of the regular least squares, in cases where that $Y\left(t\right)Y{\left(t\right)}^{T}$ is singular or nearly singular [2]. Finally, the following Simulink configurations have to be done to apply the proposed CNN classification:

- Solver type Fixed-step,
- Solver ode1 (Euler’s method [47]),
- Step size 0.5 with holding final value,
- Initial condition of CNN cells: initial state is zero.

Moreover, we are using Algorithm 2 to train our model and Equation (10) to calculate the performance accuracy of the training:

(11) $$\begin{array}{c} Accuracy=\frac{Number\;of\;correctly\;classified\;samples}{Total\;number\;of\;test\;samples}. \end{array}$$

**Algorithm 2** The learning algorithm of the CNN.
1:**Input:** Features of physiological signals  2:**Output:** CNN Templets and $A_{global}$  3:**Generate** CNNs Templates  4:**Set**$A_{global}$ to 0  5:**for**n=50,100,150,1000,1500**do**  6:    **for** β=0.1,0.01,0.001,0.0001,0.00001 **do**  7:        **for** i=1,…,10 **do**  8:           **Using** the $i^{th}$ learning dataset  9:           **Run** $SimulinkModel_{B}$  10:           **Read** *Y* from $SimulinkModel_{B}$  11:           **Using** *Y* and *g* 12:           **Estimate** $A_{global}$ using Equation (10)  13:           **Using** the $i^{th}$ learning dataset with the estimated $A_{global}$  14:           **Run** SimulinkModel B  15:           **Read** the emotion states from $SimulinkModel_{B}$  16:           **Calculate** the Accuracy  17:           **Set** the Accuracy to Accuracy repository(*i*)  18:        **end for**  **Set** the Global Parameter(n,β) = the average of the Accuracy repository  19:    **end for** 20:**end for** 21:**Select** the best CNN configuration from the Global Parameter  


#### 4.5.2. Testing Phase

After the CNN templates and $A_{global}$ is computed, Algorithm 3 is used to test our model.

**Algorithm 3** The testing algorithm of the CNN.
1:**Input:** Features of physiological signals  2:**Output:** The emotional state  3:**Using** The trained CNNs  4:**Run**$SimulinkModel_{B}$  5:**Read** The emotionl state from $SimulinkModel_{B}$


## 5. Obtained Results

This section discusses and comments on the performance results obtained by the proposed system. In order to benchmark our model, we selected the following four well-known classification concepts: (a) SVM with radial basis function (RBSVM) [48], (b) Naive Bayes classifier (NB) [49], (c) k-nearest neighbors (KNN) [50] and (d) Artificial Neural Network (ANN) [51]. Table 2 illustrates the configuration parameters of the selected classifiers including the proposed CNN.

Moreover, four performance measures are considered: Accuracy [52], Specificity, Precision and Recall [53] are calculated to give a full evaluation for the performance of our proposed system. In order to obtain valid results and the reliability of the proposed system in the same/different environment, the performance measurements are applied on three levels.

### 5.1. Overall System Performance While Using the Reference Database MAHNOB

The data generated from the subjects of MAHNOB dataset are used to evaluate our classification model in a subject independent evaluation. The data of EDA, ECG and ST sensors are divided into 70% for training and 30% for testing. The training and test sets are split from different subjects to ensure the independence between both sets.

In order to validate the combinations of different sensors, Table 3 presents the recognition accuracy based on different combinations of sensors. The results show that involving EDA, ECG and ST signals improves the overall performance. Therefore, we will focus on the performance using these signals (EDA, ECG and ST) in the following evaluation.

Moreover, Table 4 shows the classification performance results (Accuracy, Specificity, Precision and Recall) obtained for all considered classifiers.

The best performance is reached by our proposed CNN classifier, which exhibits a subject-independent accuracy value of 89.38%, a specificity value of 97.5%, a precision value of 92.11% and a recall value of 87.5%.

### 5.2. Overall Performance Evaluation While Using Both Training and Testing Data from Our Experiment

The physiological data from six subjects collected by appropriate sensors in our experiment are considered here for a further evaluation of our model. Similarly to the previous evaluation, the data are divided into 70% for training and 30% for testing. The training and test sets are selected from different subjects. Table 5 illustrates the performance results obtained. It can be seen that our CNN classifier achieved the best performance (81.88% accuracy). However, this performance is weaker when compared to the performance obtained in the previous evaluation where MAHNOB provides both training and testing data. One main reason can explain this weaker performance of our experiment when compared to the previous evaluation: the total number of subjects in our experiment is too small, just six (because it was expensive to involve many subjects). Especially for a good training, a sufficient large number of training data samples is needed. We have wished several subjects in the ranges of some dozens (at least 30 to 60). Overall, to get good statistics, a large sample set is recommended.

### 5.3. The Overall Performance Using the MAHNOB Reference Database for Training and Data from Our Experiment for Testing

In order to ensure the generalization of our proposed system, it is important to train the system on one environment (MAHNOB dataset) and test it on another environment (data from our experiment). Here, we did use all the data from MAHNOB dataset for training and the data from our experiment for testing. Table 6 shows the performance of our model as obtained for the six subjects individually before using our proposed automatic calibration model. Overall, one can see that the performance is very low and classifiers do not do better than a random choice. The best accuracy performance over all subjects is 57.19% and has been reached by our proposed CNN model.

Many environment parameters might lead to the low performance such as difference in sensors’ brands (between training and testing), elicitation materials, subject, gender, age, etc. Furthermore, calibrating the signals of a new subject before classification might significantly increase the overall classification performance. Table 7 illustrates the improvement in the classification accuracy for each subject after the calibration of the subject data using the proposed automatic calibration model. Table 8 presents a classification performance comparison between the proposed CNN before and after using the automatic calibration model. We can see that the performance accuracy of the the proposed CNN model has increased by 13.86% and reached 71.05% after involving the calibration model.

## 6. Discussion

In this paper, we have proposed a novel subject-independent emotion recognition system that shows promising results. One of the major contributions in this paper is the proposed CNN classifier. The classifier is applied on MAHNOB dataset first, just to show how the proposed classifier overcomes the standard classifiers as KNN, ANN, NB and SVM. Table 4 shows the promising performance measures of our CNN classifier using the MAHNOB dataset. However, SVM performs better than KNN, NB and ANN with 77.5, 95, 82.86 and 72.5 for accuracy, specificity, precision and recall, respectively. In contrast to that, our CNN model performs clearly better with 89.38, 97.5, 92.11 and 87.5 for accuracy, specificity, precision and recall, respectively. This can be explained because of the following reasons: (**a**) the nature of emotion recognition is a highly nonlinear dynamical system. Therefore, the history of inputs might affect the outputs. Thus, the used model should have a memory that considers the history of its inputs; this is considered by our CNN model; (**b**) because of the high parallelism of the CNN processor, this makes our CNN model a real-time model than can be implemented on embedded platforms easily [54]; and (**c**) using the paradigm of CNN for classification purposes showed promising results in the state-of-the-art [55,56].

Moreover, regarding our proposed dynamic calibration, Table 6 and Table 7 show the advantage of such a calibration module when we tried to train our classification model using the MAHNOB dataset and test it using our experiments. Table 6 still proves that, even without calibration, the CNN classifier performs well and still has a better accuracy compared to KNN, NB, and ANN, which is 57.19 in average. However, Table 7 shows the advantage of such a calibration module when we tried to train our classification model using the MAHNOB dataset and test it using our experiments after calibration. It lists the accuracy measurements for all six subjects with an accuracy of 71.05 on average. The calibration module improved the overall accuracy performance by more than 13%. This improvement can be explained due to the nature of the calibration module, which emulates the nature of human emotions that might be represented in shifted measurements values using different types of sensors. We believe that having a universal model that can recognise human emotions is highly required for different applications. Therefore, our proposed dataset has been built to make sure that a universal model for human emotion recognition is possible. This was the major research question we tried to answer.

Concerning Wearable Electrocardiogram (ECG) sensors, they cannot be considered nowadays as they are very intrusive sensors. However, due to recent innovations, ECG sensors have become available in the form of ”plaster” sensors that are less intrusive [57]. Additionally, in [58], they do propose a fully-wearable medical garment for mobile monitoring of cardiac biopotentials from the wrists or the neck with minimum restriction to regular clothing habits.

## 7. Conclusions

We presented a subject-independent emotion recognition system using physiological signals (EDA,ECG,ST) that can recognize four different emotions robustly. The proposed system is evaluated by involving a benchmark database and an emotion elicitation experiment using short video clips. The classification results of CNN (benchmarked with other state-of-the-art machine learning methods) show a significant improvement of the accuracy.

Furthermore, we found out that the subject independent human emotion recognition is one of the most challenging problems in the field of Machine Learning. This problem exacerbates when the proposed emotion recognition system should perform robustly on different environment where subjects, elicitation materials and physiological sensors brand/factories are different.

To address this challenge, we could show that our automatic calibration model could take a step towards a global subject independent emotion recognition system by improving the performance of the recognition significantly. A considerable aspect for the future is to improve the calibration model and enhance the features’ extraction approaches in order to improve the overall performance. 

## Figures and Tables

**Table 1:** Literature review on emotion recognition using physiological and speech signals.

Ref. No.	Signals	Features	Classifiers	Emotion Parameters	Stimuli	No. of Subjects	Accuracy in %
[11]	EMG ECG EDA RSP	Statistical, Energy, Sub band Spectrum, Entropy	Linear Discriminant Analysis	Joy, Anger, Sad, Pleasure	Music	3 , MITdatabase	95 (Subject-Dependent) 70 (Subject-Independent)
[14]	EDA HR ST	No specific features stated	KNN, Discriminant Function Analysis, Marquardt backpropagation	Sadness, Anger, Fear, Surprise, Frustration, Amusement	Movies	14	91.7 (Subject-Dependent)
[18]	EMG EDA BVP ECG RSP	Running mean Running standard deviation Slope	NN	Arousal, Valance	IAPS (Visual Affective Picture System)	1	96.58 Arousal 89.93 Valence (Subject-Dependent)
[12]	ECG	Fast Fourier	Tabu Search	Joy, Sadness	Movies	154	86 (Subject-Independent)
[20]	EDA HR	No specific features stated	fuzzy logic	Stress	Hyperventilation Talk preparation	80	99.5 (Subject-Independent)
[19]	BVP EMG ST EDA RSP	Statistical Features	SVM, Fisher LDA	Amusement, Contentment, Disgust, Fear, Sad, Neutral	IAPS	10	90 (Subject-Dependent)
[9]	EMG EDA ECG BVP ST RSP SPEECH	Statistical Features, BRV, Zero-crossing, MFCCs	KNN	Arousal, Valance	Quiz dataset	3	92 (Subject-Dependent) 55 (Sub Independent)
[21]	EDA HR EMG	No specific features stated	HMM	Arousal, Valance	Robot Actions	36	81 (Subject-Dependent) 66 (Subject-Independent)
[13]	EDA ECG ST	Statistical Features average power SCL SCR	CNN	Arousal, Valance	Movies	10	82.35 (Subject-Independent)

EMG: Electromyography; ECG: Electrocardiography; EDA: Electrodermal Activity; RSP: Respiration; ST: Skin Temperature; EEG: Electroencephalogram; BVP: Blood Volume Pulse; HR: Heart Rate; KNN: k-nearest neighbors algorithm; SVM: Support vector machine; HMM: Hidden Markov Model; ANN: Artificial Neural Network; CNN: Cellular Neural Network.

**Table 2:** The configuration parameters of the involved classifiers.

Classifier	Type	Parameters
RBSVM [48]	C-SVC	KernelType= radial basis function, eps= 0.001, gamma= 0.0001
NB [49]	NaiveBayes -k	UseKernelEstimator= True
KNN [50]	Default	k=2,Distance=euclidean
ANN [51]	Multilayer Perceptron	hiddenlayer=20,learningRate=0.1,momentum=0.2
CNN	Echo State	n=150,β=0.0001

**Table 3:** The recognition accuracy in % with respect to different signals combinations.

	Physiological Sensor	KNN	NB	ANN	SVM	CNN
Single sensor	ECG	61.2	53.58	53.92	62.91	56.41
	EDA	63.73	53.1	60.32	68.4	75.34
	ST	33.12	35.7	42.64	41.8	42.6
Multi sensors	EDA + ECG	71.12	55.4	60.78	72.64	83.43
	ST + ECG	68.45	54.53	55.86	70	68.63
	ST + EDA	69.13	55.34	58.43	69.64	78.5
	ST + EDA + ECG	76.88	56.88	62.5	77.5	89.38

**Table 4:** Performance measures in percentage while using the reference database MAHNOB for both training and testing data (subject-independent evaluation).

Measure	KNN	NB	ANN	SVM	CNN
Accuracy	76.88%	56.88%	62.5%	77.5%	89.38%
Specificity	95%	86.67%	85.84%	95%	97.5%
Precision	81.82%	57.9%	57.5%	82.86%	92.11%
Recall	67.5%	55%	57.5%	72.5%	87.5%

**Table 5:** Performance measures in percentage while using our experiment for both training and testing data (subject-independent evaluation).

Measure	KNN	NB	ANN	SVM	CNN
Accuracy	56.25%	25.63%	45.63%	71.88%	81.88%
Specificity	80%	72.5%	83.34%	89.17%	95%
Precision	50%	25%	42.86%	67.5%	82.86%
Recall	60%	27%	37.5%	67.5%	72.5%

**Table 6:** The performance accuracy in percentage while using the MAHNOB reference database for training and data from our experiment for testing (**without calibration model**).

Subject	KNN	NB	ANN	SVM	CNN
Subject1	30.54%	26.43%	31.21%	14.31%	58.76%
Subject2	44.15%	22.23%	30.76%	20.87%	60%
Subject3	32.25%	13.15%	34.89%	25.90%	54.38%
Subject4	29.64%	19.22%	28.33%	20.33%	57.5%
Subject5	29.90%	25.21%	12.85%	22.58%	59.38%
Subject6	27.45%	10.89%	25.07%	17.32%	53.13%
**All Subjects**	32.33%	19.53%	27.18%	20.22%	**57.19%**

**Table 7:** The performance accuracy in percentage while using the MAHNOB reference database for training and data from our experiment for testing (**using calibration model**).

Subject	KNN	NB	ANN	SVM	CNN
Subject1	44.58%	31.88%	55.63%	48.54%	70.63%
Subject2	53.93%	29.12%	58.38%	50.82%	81.26%
Subject3	48.62%	40.19%	60.45%	42.73%	71.88%
Subject4	35.55%	27.45%	33.77%	44.21%	72.5%
Subject5	24.29%	30.83%	22.36%	43.16%	63.13%
Subject6	25.23%	23.28%	30.83%	26.87%	66.88%
**All Subjects**	38.7%	30.46%	43.57%	42.73%	**71.05%**

**Table 8:** The performance measures of the CNN model in percentage before and after involving the calibration model.

Measure	CNN without Calibration Model	CNN with Calibration Model
Accuracy	57.19%	**71.05**%
Specificity	84.31%	**89.87**%
Precision	55.86%	**69.84**%
Recall	59.59%	**70.42**%
